# Enhanced UV-B photoprotection activity of carotenoids from the novel *Arthrobacter* sp. strain LAPM80 isolated from King George Island, Antarctica

**DOI:** 10.1016/j.heliyon.2024.e41400

**Published:** 2024-12-21

**Authors:** Beatriz Vivian Paredes Contreras, Alane Beatriz Vermelho, Livia Casanova, Claudia de Alencar Santos Lage, Caren Leite Spindola Vilela, Veronica da Silva Cardoso, Luis William Pacheco Arge, Janine Simas Cardoso-Rurr, Sulamita Santos Correa, Felipe Raposo Passos De Mansoldo, Maria Cristina Pinheiro Pereira Reis-Mansur, Eikon Alves da Silva, Júnia Schultz, Alexandre Soares Rosado

**Affiliations:** a Laboratory of Molecular Microbial Ecology, Microbiology Institute, Federal University of Rio de Janeiro, Rio de Janeiro, Brazil; b BioInovar Laboratory, Microbiology Institute, Federal University of Rio de Janeiro, Rio de Janeiro, Brazil; c Laboratory of Radiations in Biology, Institute of Biophysics Carlos Chagas Filho, Federal University of Rio de Janeiro, Rio de Janeiro, Brazil; d Laboratory of Molecular Genetics and Plant Biotechnology, Federal University of Rio de Janeiro, Rio de Janeiro, Brazil; e Biological and Environmental Science and Engineering Division, King Abdullah University of Science and Technology, Makkah, 23955, Saudi Arabia

**Keywords:** Carotenoids, Antarctica, Ultraviolet radiation, *Actinomycetota*

## Abstract

Antarctica's harsh environmental conditions, characterized by high levels of ultraviolet (UV) radiation, pose challenges for microorganisms. To survive in these extreme cold regions with heightened UV exposure, microorganisms employ various adaptive strategies, including photoprotective carotenoid synthesis. Carotenoids are garnering attention in the skin health industry because of their UV photoprotection potential, given the direct relationship between UV exposure and skin burns, and cancer. Also, there is a growing demand for natural and environmentally friendly photoprotectors, such as microbial-based products, in opposition to synthetic photoprotective agents with known adverse effects.

In this study, we assessed the carotenoid-producing abilities of *Actinomycetota* strains from Antarctic Peninsula soils and the photoprotective carotenoid action on UV irradiation resistance. Among 20 evaluated strains, one exhibited significant carotenoid production and it was identified through genomic analysis as a likely novel *Arthrobacter* sp. strain, LAPM80. This strain's genome revealed the presence of genes coding for the biosynthesis of decaprenoxanthin C50 carotenoid. The LAPM80 strain exhibited enhanced resistance against UV-B irradiation, correlating with increased total carotenoid production in its stationary growth phase. Chemical characterization of the carotenoid extract identified major components as C50 carotenoids, probably decaprenoxanthin and/or sarcinaxanthin. Scanning electron microscopy revealed minimal surface changes in bacteria during carotenoid-rich phase after UV-B irradiation exposure.

These findings highlight the likely ability of LAPM80 strain's C50 carotenoids to improve UV-B iiradiation resistance, indicating their potential for developing natural photoprotective compounds for the dermo-cosmetic industry.

## 1 Introduction

Synthetic pigments have extensive applications across various sectors, such as cosmetics, paints, and plastics. They dominate the market due to their cost-effectiveness in large-scale production [1]. However, their predominant use of nonrenewable petrochemicals [2] can lead to the generation of toxic chemical waste, posing environmental and human health concerns. Consequently, a growing demand for environmentally friendly pigments is expected to drive market growth. Although natural pigments derived from plants are a potential alternative, their production requires land and is subjected to variations based on climate. Moreover, the extraction process is costly and time-consuming [1]. Commercially explored insect pigments, such as carmine from cochineal, are considered natural alternatives, but they pose several ethical concerns and disadvantages [3].

Microorganisms are also a promising source of natural pigments, offering eco-friendly, nontoxic, and biodegradable alternatives. These characteristics make microbes a vital reservoir for pigment production, with wide-ranging biotechnological applications across various industries, including cosmetics, pharmaceuticals, textiles, medicine, food, and nutrient supplements. Microbial pigments, which are synthesized by fungi, yeasts, microalgae, and Bacteria and Archaea domains, encompass various biomolecules (e.g., carotenoids, flavins, phenazines, chlorophylls, violaceins, and melanins), reflecting the extensive diversity of pigments. This diversity underlines the significant potential of microbial pigments in industrial applications [1,4,5].

Bacteria, a well-studied group of microorganisms, produce pigments through secondary metabolism, attracting increased interest in their economic sustainability [118]. Bacterial pigments offer numerous benefits, including short life cycles that reduce production time, and the potential for sustainable and eco-friendly production through bioprocess engineering. Advancements in metabolic engineering, recombinant biology, synthetic biology, and omics sciences have improved pigment expression and production for commercial use [4]; [113]; [5]. An illustrative case is blue indigo production by recombinant bacteria [2]. Additionally, metabolic engineering has resulted in the overproduction of C50 carotenoids in *C. glutamicum* [116].

Microbial pigments have bioactive properties, opening up new commercial possibilities beyond color utilization. These properties include immunosuppressive, anticancer, antimicrobial, antioxidant, and anti-inflammatory activities [1,6]. Other related functions include energy storage [1,7] and ultraviolet (UV) protection [5,8]. About the last property, many bacterial pigments, typically associated with photosynthesis and adaptation to extreme environments, protect against UV damage [9].Also, various pigmented bacterial groups from different environments, such as *Virgibacillus salaries* strain 19.PP.Sc1.6, *Micrococcus luteus*, *Cryseobacterium pallidum*, *Cryseobacterium* spp., and *Kocuria turfanensis*, have been reported to produce UV-protective pigments [10,11]. Carotenoids, prominent among the UV-protective pigments, offered defense against photo-oxidative damage [12,13], and shield proteins, lipids, and DNA from oxidative stress induced by UV irradiation [[14], [15], [16]].

Extreme solar radiation environments, such as those in Antarctica, are ideal for isolating bacteria that produce carotenoids with photoprotective functions. Antarctica experiences high radiation intensities due to frequent ozone depletion in late spring ([112]; [111]; [114]), so Antarctic microorganisms need to endure intense UV radiation for survival [117]; [121]. Notably, many Antarctic microorganisms that live under harsh UV conditions, not only them but also microorganisms from solar saltern ponds for instance, mainly produce rare long-chain carotenoids such as C50, which are likely crucial for UV survival [17,60]; [119]. Within the terrestrial bacterial community of Antarctic, the *Actinomycetota* group is dominant [122], with species such as *Arthrobacter* able to synthesize carotenoids, enhancing their UV resistance [12]. Moreover, our research group has identified that the Antarctic *Microbacterium* sp. LEMMJ01 produces carotenoids with UV-protective properties, incorporating them into sunscreen formulations [52,88]. However, despite these findings, many Antarctic microorganisms still require isolation and exploration in the laboratory, presenting significant untapped potential for pigment production [115].

Investigating novel Antarctic bacteria capable of synthesizing rare carotenoids with photoprotective properties holds industrial significance, especially considering the potential of UV-B rays (280–315 nm) to cause skin cancer and burns [106,107], and there is also a concern about an expected increase of UV-C rays (100–280 nm) in the future due to the depletion of the ozone layer caused by air pollution [120]. The demand for natural sunscreens is upgrowing, as chemical photoprotectors have been scrutinized in terms of their adverse effects [108,109]. This study aims to bioprospect natural carotenoid-producing *Actinomycetota* strains from Antarctica and assess the pigment's contribution to UV-B and UV-C irradiation resistance. Our study encompasses genome characterization, functional annotation, partial carotenoid characterization, and scanning electron microscopy (SEM) analysis. Additionally, the potential application of these pigments in sunscreen formulations will be explored from a future perspective.

## 2 Materials and methods

### 2.1 Bacterial strains and culture conditions

Twenty bacterial strains from the *Actinomycetota* phylum were selected for this study from the Antarctic Bacterial Collection at the Laboratory of Microbial Molecular Ecology (LEMM), Federal University of Rio de Janeiro, Brazil. These strains were isolated from soil samples collected across various locations in Admiralty Bay, King George Island, in the South Shetland Islands of Antarctica, as outlined by Silva et al. [18], with support from the Brazilian Antarctic Program (PROANTAR). The isolates (referenced in Table S1) were revived from stock cultures by inoculating 10 μL into liquid Lysogeny Broth (LB) and then incubated at 25 °C with constant shaking at 120 rpm in a thermostatic shaking incubator (CIENTEC, Model CT-712R). Subsequently, the cultures were plated on solid LB media and incubated at 25 °C to verify their purity and observe pigment production. The set temperature was previously tested in the early reactivation phase and that one guaranteed optimal growth and pigmentation of the strains in comparison with 4 °C.

### 2.2 Bioprospecting of carotenoid-producing bacteria

Carotenoid content was determined following the methods outlined by Libkind et al. [19] and Khodaiyan et al. [20], with some modifications. Briefly, the strains were cultured in triplicate in 50 mL of LB medium for 7 days at 25 °C and 120 rpm in an incubator with orbital agitation (Tecnal, TE-421). Cultures were then centrifuged for 20 min at 4 °C and 5000 rpm (Thermo Scientific, Sorvall, ST 16R), and the resulting pellets were washed twice with saline solution (0.85 % NaCl) before being centrifuged again. Carotenoid extraction was performed by adding 5 μL of DMSO per mg of wet biomass, followed by incubation in a water bath for 5 min (50 °C) and vortexing. The mixture was then centrifuged, the supernatant was collected, and the extraction procedure was repeated three times. Next, 200 μL of each sample was added in triplicate to 96-well microplates to measure the total carotenoid content (KASVI K12-096). Absorbance readings were obtained at 450 nm ([21]; Rodriguez-Amaya et al., 2024) using a SpectraMax® i3x microplate spectrophotometer (Molecular Devices), and quantification was based on the analytical curve of beta-carotene (100–1000 μg/mL). The carotenoid concentration was expressed as μg of carotenoids per mg of biomass. Finally, the strain with the highest total carotenoid yield was selected for further procedures.

### 2.3 Genomic analysis, phylogenetic correlations, and annotation for carotenoid biosynthesis

The *Actinomycetota* strain with the highest total carotenoid yield (LAPM80) was selected and incubated in 5 mL of LB medium for 48 h at 25 °C and 120 rpm. DNA was extracted following the specifications of the Wizard® Genomic DNA Purification Kit (Promega, #A1125), and the 16S rRNA gene was amplified using the bacteria-specific primers 27F (5′AGAGTTTGATCMTGGCTCAG 3′) [22] and 1492R (5′GGTTACCTTGTTACG ACTT 3′) [23]. Polymerase Chain Reaction (PCR) amplification was conducted as follows: a mixture of 12.5 μL of 2X Master Mix (EconoTaq® Plus Green #30033-2), 9 μL of sterile Milli-Q water, and 1.5 μL of genomic DNA. The PCR procedure involved thermocycling steps, starting with an initial denaturation at 94 °C for 2 min, followed by 35 cycles at 94 °C for 30 s, 55 °C for 30 s, 72 °C for 40 s, and a final extension at 72 °C for 7 min. The PCR product was verified by agarose gel electrophoresis (1.2 %), and purified using the Illustra GFX PCR DNA Kit and the Gel Band Purification Kit (GE Healthcare).

Amplification and sequencing of PCR products using the Sanger method were conducted at the Ramaciotti Centre for Genomics (University of New South Wales, Australia). The Ribosomal Database Project (RDP) program (http://rdp.cme.msu.edu/) [24] was employed to trim the received raw sequences following standard parameters. The Bioedit Sequence Alignment Editor Program (Version 7.2) [25] was used to assemble contigs, and the assembled result was compared with the RDP and NCBI nucleotide databases (http://www.ncbi.nlm.nih.gov/) using BLASTn to infer the taxonomy of the strain.

The genomic DNA of the LAPM80 strain was sent to Macrogen (Macrogen, Seoul, Korea) for whole-genome sequencing and deeper characterization. Library preparation was performed using the TruSeq DNA Nano 350-bp kit, and sequencing was conducted on the Illumina NovaSeq platform in 150-bp paired mode, following the manufacturer's instructions. The quality of the raw reads was evaluated using FastQC v.0.11.5 software [26], and Trimmomatic v.0.39 [27] was employed to eliminate low-quality adapters and bases. Reads without adapters and exhibiting good quality were utilized for genome assembly using the Unicycler assembler v.0.5.0 [28]. Subsequently, the genome quality was assessed using Quast v.5.2.0 software [29]. Then, BUSCO v.5.4.5 [30] was used to assess the completeness of single-copy orthologs. Furthermore, average nucleotide identity (ANI) calculations [31] and digital DNA–DNA hybridization (dDDH) [32] were conducted to determine the taxonomy and similarity of the strains with public databases, following the recommended parameters and settings.

The assembled genome was annotated using PROKKA v.3.0 [33]. Subsequently, the genome sequence of the strain was uploaded to the Type (Strain) Genome Server (TYGS) for whole genome-based taxonomy analysis [32]. Taxonomic information, including nomenclature, synonymy and associated literature, was obtained from the LPSN database (List of Prokaryotic Names with Standing in Nomenclature, available at https://lpsn.dsmz.de) [34]. The closest type strain genomes were determined using two complementary processes. First, the strain genome was compared against all type strain genomes available in the TYGS database using the MASH algorithm, a fast approximation of intergenomic relatedness [35], with only the 10 type strains displaying the smallest MASH distances selected. Second, an additional set of 10 closely related strains was identified using 16S rDNA gene sequences. These sequences were extracted from the strain genome using RNAmmer [36], and each sequence was subsequently BLASTed [37] against the 16S rDNA gene sequence of each of the type strains currently available in the TYGS database. This served as a proxy to detect the top 50 matching type strains (based on bitscore) and calculate precise distances using the Genome BLAST Distance Phylogeny (GBDP) approach under the “coverage” algorithm and the d5 distance formula [38]. These distances were then used to determine the 10 closest type strain genomes.

Phylogenomic analysis was conducted in the same TYGS platform. Pairwise comparisons among the genome sets were executed using the GBDP method, with calculation of distances using the “trimming” algorithm and the d5 distance formula [38]. For each genome, 100 distance replicates were computed. Furthermore, the dDDH values and their confidence intervals were estimated using the GGDC 4.0 settings [34,38]. These distances facilitated the construction of a balanced minimum evolution tree, with branch support evaluated using FASTME 2.1.6.1, including corporating Subtree Pruning and Regrafting postprocessing [39]. Branch support was derived from 100 pseudobootstrap replicates. The tree was rooted at its midpoint [40], visualized using PhyD3 [41], and further refined using iTOL v.4.261 [42]. Species clustering based on type strains was conducted using a 70 % dDDH radius, following established methods [32].

Alignment comparisons with the KEGG database were conducted using GhostKOALA [43], and the curated genome was analyzed for secondary metabolic clusters with antiSMASH [44]. Functional annotation was achieved using Prodigal v.2.6.3 [45] in its default single mode and DIAMOND (v2.1.9.163) [46] for BLASTp searches against the NCBI nonredundant (NCBI-nr) protein sequences database.

### 2.4 Growth curve and carotenoid production

A bacterial suspension of the selected strain was prepared and standardized, using a saline solution (0.85 % NaCl), from a Petri plate with bacterial growth. The suspension was quantified by measuring the optical density at 630 nm (OD_630_), corresponding to 0.1 (∼1.5 × 10^8^ CFU/mL, equivalent to 0.5 McFarland standard), and used as the inoculum in 75 mL of LB medium [[47], [48], [49]]. Incubation was conducted in an agitation incubator (Tecnal, TE-421) for172h at 25 °C and 120 rpm. The experiment was conducted in triplicate, and aliquots were collected at 15 different time points. For each time point, 200 μL aliquots were deposited into 96-well microplates (Kasvi K12-096) in triplicate to determine the OD_630_ along the growth curve using the SpectraMax® i3x instrument (Molecular Devices).

Additional aliquots collected in microtubes at the 15 different time points were used to evaluate carotenoid production. The extraction and quantification of total carotenoids followed the procedures described in the “Bioprospecting of carotenoid-producing bacteria” section, with adaptations. Aliquots were centrifuged for 5 min at 4 °C and 14000 RCF (Centrifuge 5430, Eppendorf), and pellets were washed twice with saline solution before being centrifuged again. Subsequently, 600 μL of DMSO was added to the wet biomass, followed by incubation in a water bath for 5 min (50 °C) and homogenization. Subsequently, the samples were centrifuged, and extraction procedures were repeated three times. Finally, 200 μL of each sample was added to 96-well microplates (Kasvi K12-096) in triplicate, and absorbance readings were obtained at 450 nm using a SpectraMax® i3x instrument (Molecular Devices). The carotenoid concentration was expressed as μg of carotenoids per mL of DMSO extract. Quantification was based on the analytical curve of beta-carotene (100–1000 μg/mL).

### 2.5 UV radiation study

The selected strain was tested for resistance to UV-B and UV-C irradiation. The bacterium was cultured in 75 mL of LB medium at 25 °C and 120 rpm until it reached the logarithmic and stationary growth phases, separately (time determined in the previous section). Cultures were centrifuged for 5 min at 4 °C and 8000 rpm (Thermo Scientific, SORVALL, ST. 16R). Bacterial cells were washed twice in saline solution (0.85 % NaCl) and resuspended. The suspensions were adjusted to reach an OD_630_ of ∼0.6 and added to sterile glass Petri dishes. Bacterial suspensions were exposed to 0, 4, 8 e 16 kJ/m^2^ of UV-B irradiation doses using an ultraviolet lamp with an emission peak at 312 nm (15 W with filter; model VL-215 LM (VilberLoumart)), and exposed to 0, 50, 100, 200 e 300 J/m^2^ of UV-C irradiation doses using a germicidal lamp with an emission peak at 254 nm (G15T8 15 W; Sankyo Denky). The experiment was conducted using three biological replicates. Total dose of UV-B and UV-C irradiation were determined using a VLX-3W model dosimeter (VilberLourmat) coupled with a 312 nm photocell (Series 21–216), and a 254 nm photocell (Series 11–102202), respectively. The UV-B irradiation dose rate in the treatment was 15 J/m^2^/s and the UV-C dose rate was 2 J/m^2^/s. UV irradiation doses were calculated by multiplying the measured intensity by the irradiation time (seconds).

Following UV irradiation, the irradiated aliquots were serially diluted (1:10^1^–1:10^7^). Subsequently, 10 μL of each dilution was dropped onto LB agar plates in triplicate, and the plates were incubated for 2–5 days at 25 °C. Colony-forming units (CFUs) of irradiated samples (N) were estimated and divided by CFUs of nonirradiated samples (N0) to determine the survival fraction (N/N0). The values were represented by their mean and standard deviation. Methodological considerations were adapted from previous studies [[50], [51], [52], [53]] with some adaptations, including the use of a few drop volume in agar plates after dilution [54,55]. The Mann–Whitney test (one-tailed) was used to assess statistical significance. Additionally, the same procedures were followed to compare the UV irradiation resistance of the selected strain with a reference strain, *E. coli* wild-type K12A15, during the stationary growth phase.

### 2.6 Partial carotenoid chemical characterization

The carotenoid extract of the selected strain was analyzed using high-performance liquid chromatography with diode-ray detection (HPLC-DAD) in an Agilent 1260 Infinity II Chromatograph. DMSO was gradually evaporated from the extract upon adding water at 50 °C. Subsequently, the sample was lyophilized using an SL-404 lyophilizer (Solab) and resuspended in a mixture comprising 400 μL of Milli-Q water, 550 μL of acetonitrile, and 150 μL of isopropanol. The resuspended solution was filtered through polyamide filters (NY 13 mm, 0.22 μm) (Unifil). Chromatographic analysis was conducted on an RP-18 column (length of 25 cm, inner diameter of 4.6 mm, and particle sizer of 5 μM; Kromasil) maintained at 30 °C. Samples were injected into the column and eluted using a gradient of Milli-Q water containing 0.1 % formic acid (Solvent A), and acetonitrile/isopropanol (7:3) containing 0.1 % formic acid (Solvent B). The gradient profile was as follows: 0–5 min (5%–50 % B), 5–20 min (50%–100 % B), 20–45 min (100 % B), and 50–55 min (100%–5% B). A total of 50 μL of the sample was injected, and the flow rate was maintained at 1 mL/min. Detection was conducted at 450 nm, and total carotenoid identification was based on their UV/VIS absorption spectra (200–640 nm).

High-performance liquid chromatography with diode-array detection coupled with tandem mass spectrometry (HPLC-DAD-MS/MS) was conducted on a Nexera X2 (Shimadzu) liquid chromatography system coupled to a Maxis Impact (Bruker) mass spectrometer equipped with an APCI ion source and a Q-TOF analyzer. Following the removal of DMSO and lyophilization of the extract, the total carotenoid extract of the best isolate was solubilized in 1 mL of acetone and diluted to a concentration of 10 mg/mL. Chromatographic runs were performed on a Hypersil C18 column (150 × 1 mm, 3 μm; Thermo Fisher Scientific) maintained at 25 °C. The mobile phase comprised Milli-Q water (Solvent A) and acetone (Solvent B) at a flow rate of 0.2 mL/min, with the following gradient profile: 0–8 min (75–95 % B), 8–14 min (95 % B), 14–16 min (95–100 % B), 16–24 min (100 % B), 24–24.1min (75 % B), 24.1–25 min (75 % B). The injection volume was 10 μL, and the analysis was monitored between 330 and 700 nm. The Q-TOF mass spectrometer was operated in positive-ion mode with the following parameters: capillary temperature = 200 °C, flow rate of auxiliary gas = 4 L/min, evaporation temperature = 400 °C, and corona current = 8000 V. Nitrogen served as a collision and auxiliary gas in the source. The mass range analyzed was from *m*/*z* 50 to 1200. Internal calibration was achieved using 100 μM of sodium formate in water/acetonitrile (1:1). Data-dependent acquisition (AutoMS) was conducted with the isolation/fragmentation of three precursors per cycle. The data were processed using MZmine software (Version 3.0), and the elemental composition of the detected compounds was determined considering mass errors below 10 ppm.

### 2.7 Scanning electron microscopy analysis

SEM was used to analyze the effects of UV-B irradiation on the selected strain's surface. One sample collected during the logarithmic growth phase was subjected to 0 and 4 kJ/m^2^ doses of UV-B irradiation. Another sample harvested during the stationary growth phase was subjected to 0 and 6 kJ/m^2^ doses of UV-B irradiation. These doses were determined using the graph from the “UV Radiation Study” section, where the bacterium presented 100 % and 1 % survival rates under UV-B irradiation. The irradiated samples were stored in Eppendorf tubes and centrifuged for 5 min at 4 °C and 14000 RCF (Centrifuge 5430, Eppendorf). Bacterial cells were processed as follows: (i) fixation in 2.5 % glutaraldehyde in 0.1 M cacodylate buffer for 1 h at room temperature; (ii) three washes in 0.1 M cacodylate buffer; (iii) adhesion to poly-L-lysine-coated glass coverslips; (iv) postfixation in 1 % osmium tetroxide in 0.1 M cacodylate buffer for 1 h; (v) three washes in 0.1 M cacodylate buffer; (vi) dehydration in a graded ethanol series (30–100 %); (vii) critical-point drying with CO_2_; and (viii) gold coating during metallization. SEM mode was used to characterize sample morphology and microstructures (Carl Zeiss EVO MA10).

## 3 Results and discussion

### 3.1 Bacterial strains and bioprospecting of carotenoid-producing bacteria

**Fig. 1:**
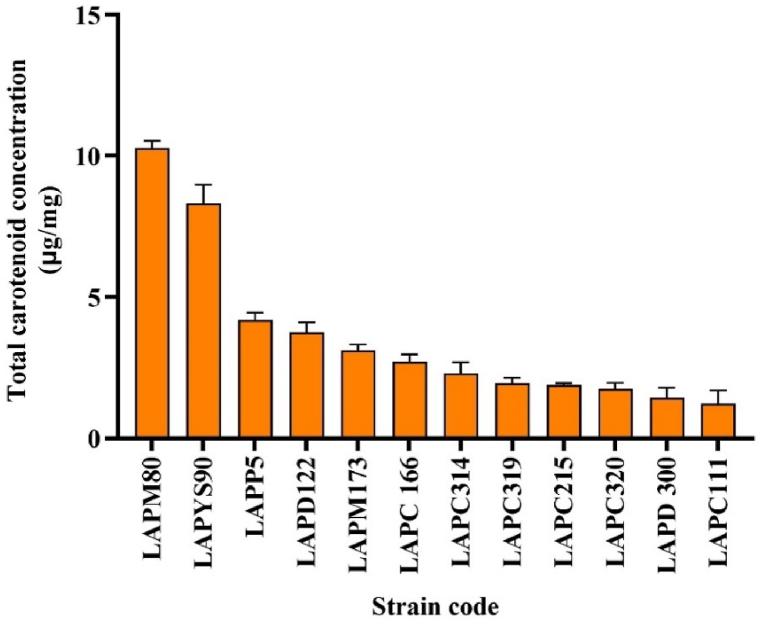
Carotenoid concentration in bacterial isolates from Antarctica. The carotenoid concentration was expressed as μg of carotenoids per mg of biomass.

Among the 20 reactivated isolates, 12 strains showed satisfactory growth and pigmentation following reactivation in the solid LB medium, presenting several shades of yellow (LAPP5, LAPM80, LAPYS90, LAPC111, LAPC215, LAPC314, LAPC319, and LAPC320) and orange (LAPD122, LAPC166, LAPM173, and LAPD300). During the bioprospecting of carotenoid-producing bacteria, the LAPM80 isolate showed the highest total carotenoid content compared to other strains (Fig. 1) and was selected for further studies. All strains were submitted to the same conditions of growth and nutrients, then differences in their carotenoid concentrations could be due to genetic variability of each strain, specific metabolic pathway of each one, and environmental stressors that they were exposed in the local of isolation.

The high total carotenoid content in LAPM80 may contribute significantly to its oxidative damage resistance. The antioxidant capacity of carotenoids tends to increase with carotenoid concentration, as demonstrated in various *in vitro* studies [[56], [57], [58]]. Therefore, the high carotenoid concentration in this isolate likely provides enhanced resistance to UV-induced photo-oxidative stress.

### 3.2 Genomic analysis, phylogenetic correlations, and annotation for carotenoid biosynthesis

The LAPM80 strain showed the highest concentration of carotenoid content and was taxonomically characterized using 16S rRNA gene sequencing. Its sequence (NCBI accession number PP708905) was compared with those available in the RDP and NCBI databases, and exhibited a close relation to the *Arthrobacter* genus, aligning with *Arthrobacter* sp. strain KOPRI 25481 (0.950 similarity score in RDP) and *Arthrobacter* sp. strain E3-12 (98.85 % identity in NCBI). This genus is frequently found in the soils of Maritime Antarctica [[59], [60], [61], [62]].

**Fig. 2:**
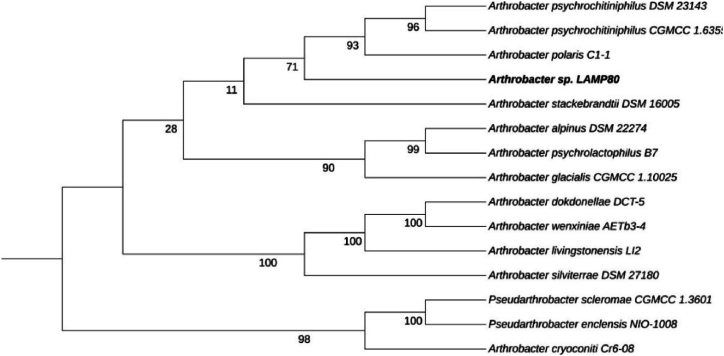
Tree inferred using FastME 2.1.6.1 [39] from GBDP distances estimated from genome sequences. Branch lengths are scaled according to the Genome BLAST Distance Phylogeny (GBDP) distance formula d5. The numbers above the branches are GBDP pseudo-bootstrap support values > 60 % from 100 replications, with an average branch support of 82.2 %. The tree was rooted at the midpoint [40], and the resulting Newick tree file was modified using Itol v.4.261 [42].

A summary of the assembly statistics and genome information is presented in Table S2. The draft genome of the LAPM80 strain was constructed with high-quality sequences, generating 68 contigs, a total genome length of 4034.042 bp, GC content of 62.80 %, and 3.699 codifying sequences. Based on taxonomic analysis, the strain was phylogenomically closest to *Arthrobacter polaris* C1-1 (dDDH = ∼22.4 %, ANIb = ∼80.89 %). The dDDH value was lower than the cutoff for species delineation (70 %), and the ANIb value was below the accepted threshold for species delimitation (95%–96 %). Thus, these results suggest that the selected strain represents a potential new species within the genus *Arthrobacter,* referred to as *Arthrobacter* sp*.* strain LAPM80*.* However, further genomic studies are required to confirm its status as a new species. In addition, the genome-inferred tree of the LAPM80 strain is shown in Fig. 2.

Using antiSMASH platform, carotenoid production was predicted from the genome of *Arthrobacter* sp. strain LAPM80 with 21 % similarity. Alignment comparison with the KEGG database identified the enzyme phytoene synthase in the carotenoid pathway of the strain. Additionally, Prokka annotation revealed phytoene dehydrogenase, lycopene elongase, and C50 carotenoid epsilon cyclase. Moreover, functional annotation of the Prodigal–Diamond–NCBI-nr pipeline revealed the presence of enzymes such as phytoene synthase, phytoene desaturase, phytoene dehydrogenase, lycopene cyclase, zeaxanthin glucosyltransferase, geranylgeranyl pyrophosphate synthase, and C50 carotenoid epsilon cyclase (Table S3). Enzymes belonging to the KEGG carotenoid pathway are marked in the diagram in Fig. S1.

Geranylgeranyl pyrophosphate synthase catalyzes the conversion of farnesyl pyrophosphate (C15) to geranylgeranyl pyrophosphate (GGPP) (C20), which serves as a precursor for carotenoids [63]. Phytoene synthase is involved in the initial steps of carotenoid biosynthesis, mediating the conversion of phytoene from two molecules of GGPP (the first colorless C40 carotenoid) [64]. Phytoene desaturase facilitates the conversion of phytoene into lycopene (C40) [65]. Lycopene elongase converts lycopene to flavuxanthin (linear C50 acyclic), while C50 carotenoid epsilon cyclase mediates the conversion to decaprenoxanthin (C50 cyclic) [63]. Furthermore, phytoene dehydrogenase is considered a rate-limiting enzyme in carotenoid synthesis [66]. Lycopene cyclase aids in converting beta-carotene (C40) from lycopene [64], and zeaxanthin glucosyltransferase converts zeaxanthin (C40) to zeaxanthin diglucoside [67]. This information indicates that the LAPM80 strain can synthesize zeaxanthin, beta-carotene, lycopene, and a rare C50 carotenoid (decaprenoxanthin). In comparison, some previous bioinformatics-molecular studies in *Corynebacterium glutamicum* [65], *Micrococcus luteus* [68], and extremophilic Haloarchaea [69] have analyzed C50 carotenoid pathways.

### 3.3 Growth curve and carotenoid production

**Fig. 3:**
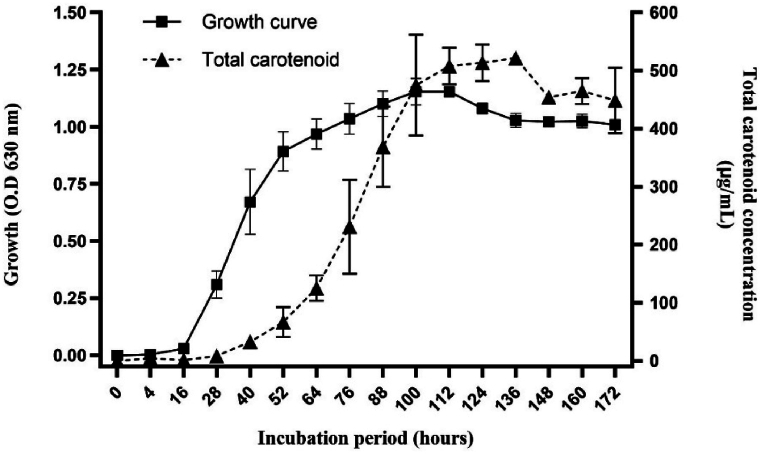
Growth curve (O.D_630_) (■) and total carotenoid production concentration (μg/mL) (▲) along 172 h of cultivation of *Arthrobacter* sp. strain LAPM80. The carotenoid concentration was expressed as μg of carotenoids per mL of DMSO extract Graph points represent the average of the readings, and error bars represent the standard deviations.

The growth curve of *Arthrobacter* sp*.* strain LAPM80 entered the logarithmic growth phase between 28 and 40 h, reaching the stationary phase at nearly 100 h (Fig. 3). Carotenoid production commenced between 28 and 40 h, corresponding to the logarithmic growth phase, and peaked between 100 and 136 h, corresponding to the stationary growth phase (Fig. 3). As observed, like secondary metabolites, usually carotenoid accumulation in microorganisms starts in the late logarithmic phase of growth [70].

Similarly, *Arthrobacter arilitensis*, derived from smear-ripened cheeses, exhibited initial carotenoid production during the logarithmic growth phase, occurring between 0 and 48 h, with peak pigment yield observed during the stationary growth phase at approximately 144 h [71]. Additionally, a similar pattern of carotenoid production in the growth phases was observed in other bacteria genera such as *Brevibacterium linens* [21], *Exiguobacterium aurantiacum* FH [72], *Dietzia natronolimnaea* HS-1 [70], and *Citricoccus parietis* [73].

### 3.4 UV radiation study

**Fig. 4:**
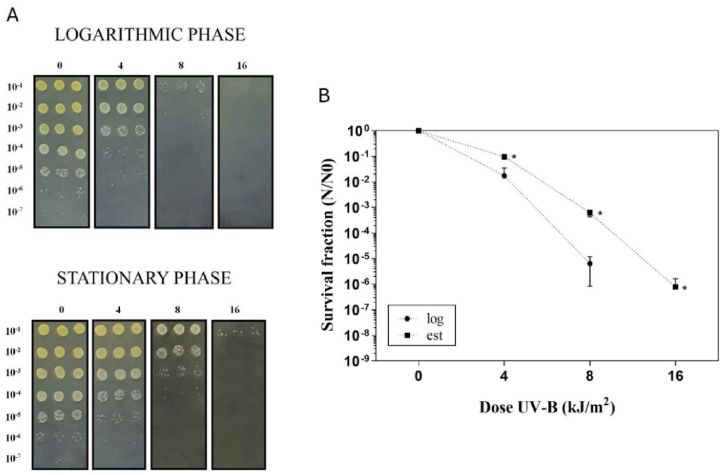
UV-B irradiation survival of *Arthrobacter* sp. strain LAPM80. (A) CFUs after exposing the strain in the growth phases to 0, 4, 8, and 16 kJ/m^2^ doses of UV-B irradiation (312 nm). Bacteria were serially diluted (1:10^1^–1:10^7^) and dropped onto LB agar plates. (B) Survival fraction of LAPM80 strain in the logarithmic phase (●) and stationary phase (■) after exposure to the UV-B doses of irradiation. Error bars represent the standard deviation of three biological replicates. Asterisks indicate a significant difference (p < 0.05) between the survival of both growth phases, assessed using a one-tailed Mann–Whitney significance test.

*Arthrobacter* sp. LAPM80 cells were evaluated for UV-B (312 nm) and UV-C (254 nm) irradiation resistance in both logarithmic and stationary growth phases, characterized by low and high pigment production, respectively. Regarding the UV-B irradiation test, the LAPM80 strain exhibited greater significant resistance to UV-B in the stationary growth phase than the logarithmic growth phase (Fig. 4). This significant difference coincided with an increase in the total carotenoid fraction. Santos et al. [50] stated that UV-B irradiation can cause photo-oxidation by generating reactive oxygen species (ROS) and oxidizing proteins and lipids. Additionally, they proposed that UV-B-induced oxidative damage is minor than the effects of UV-A irradiation, but more significant than those of UV-C irradiation. Thus, regarding the relation between UV-B and oxidative damage, the higher UV-B resistance likely linked to the maximum yield of carotenoid production could be associated with a greater protective capacity against oxidative damage conferred by elevated carotenoid concentration [[56], [57], [58]]. Moreover, the carotenoids of *Arthrobacter* sp. LAPM80 could have photoprotective properties against oxidative damage induced by UV-B irradiation. A similar pattern was obtained in a C50 carotenoid producer, where an *Arthrobacter* from the Namib Desert (Africa) presented possible bacterioruberin production and exhibited greater UV-B (302 nm) survival than non-pigmented cells. The authors noted that pigmentation may be contributed to resistance to UVB-mediated ROS damage [74]. In addition, there is a research of other carotenoid-producing bacteria that also linked UV irradiation resistance to the stationary growth phase. To illustrate, yellow carotenoids from *Pantoea stewartii* (a phytopathogen of corn) were proposed as photoprotective metabolites against UV-A irradiation in the stationary growth phase. In this phase, the wild-type strain demonstrated greater UV resistance than a colorless mutant strain, however, both strains were equally susceptible in the logarithmic phase [75].

Although some studies have investigated the UV-B irradiation resistance of pigmented *Arthrobacter* in various environments, the specific protective pigments have not been identified. For instance, *Arthrobacter agilis*, a pink strain from coastal waters (Mediterranean Sea), exhibited moderate resistance to simulated solar radiation (UV-A, UV-B, and photosynthetically active radiation) in its stationary phase. It has been suggested that carotenoids could contribute to this resistance [76]. In another study, the dark pink *Arthrobacter agilis* strain MB8-13 (Antarctic aquatic system) exhibited higher resistance to simulated solar radiation than non-pigmented isolates [77]. This increased survival of pigmented bacteria was likely due to the presence of carotenoids, with varying responses possibly because of differences in pigment structure, concentration, and location within the cell membrane. Also, an *Arthrobacter* strain from high-altitude Andean lakes (Argentina) exhibited high resistance to UV-B irradiation (312 nm) across various exposure doses. The study highlighted a challenge in researching mechanisms of UV resistance that have coincidence with the presence of secondary metabolites [78]. However, there is a report of *Arthrobacter* survival to UV-B irradiation that identified the protective pigments. *Arthrobacter psychrochitiniphilus* (defrost film, Antarctica) with decaprenoxanthin production, and *Arthrobacter agilis* (shells found in bird nests, Antarctica) with bacterioruberin synthesis showed resistance to UV-B (310 nm). Additionally, these pigments exhibited high antioxidant activity, suggesting that carotenoid identification could contribute to understand how bacteria thrive in conditions of high UV exposure in Antarctica [12].

In the case of the UV-C irradiation test, the *Arthrobacter* sp. strain LAPM80 showed no significant difference in survival between both growth phases. Despite the high carotenoid production in the stationary growth phase, it did not appear to influence protection against UV-C damage (Fig. S2), as observed for UV-B radiation. This result may be explained because UV-C rays directly interact with DNA, causing more double-strand breaks, with oxidative damage being less pronounced compared to the effects of UV-B irradiation [50]. Thus, the carotenoid extract from the LAPM80 strain is likely to have no significant aid in UV-C irradiation survival. A similar pattern was observed in a study involving a C50 carotenoid producer, where there was no significant difference in UV-C (254 nm) survival between pink-pigmented *Arthrobacter* cells (Namib Desert, Africa), possibly producing bacterioruberin and non-pigmented cells. This suggests that the pigmentation of the strain may not contribute to reducing the direct DNA/protein damage induced by UV-C radiation [74].

According to our results, many studies of UV irradiation indicated that carotenoid production is more associated to the UV-B survival, but not to the UV-C. The yellow bacterium *Cellulophaga fucicola* (sea sponges, Antarctica) with zeaxanthin, beta-cryptoxanthin, and beta-carotene synthesis, exhibited resistance to UV-B irradiation but not to UV-C, also the pigment displayed significantly high antioxidant activity [79]. The same pattern of UV survival was observed in *Arthrobacter psychrochitiniphilus* (defrost film, Antarctic), producing decaprenoxanthin and derivatives, and *Arthrobacter agilis* (shells found on bird nest, Antarctica), which had bacterioruberin and derivatives, both were more resistant to UV-B irradiation (310 nm) than UV-C (254 nm). In addition, pigments showed high antioxidant activity [12]. Same as, a yellow *Flavobacterium* sp. and an orange *Brevibacterium* sp. (mangrove soil, Brazil) were found to be more resistant to UVA-B (320–400 nm) than to UV-C (254 nm) in the exponential growth phase. The yellow pigment (zeaxanthin) and the orange one (beta-carotene and canthaxanthin) offered more protection against UVA-B than UV-C irradiation, adding the carotenoid extracts to *E.coli* suspensions [80].

**Fig. 5:**
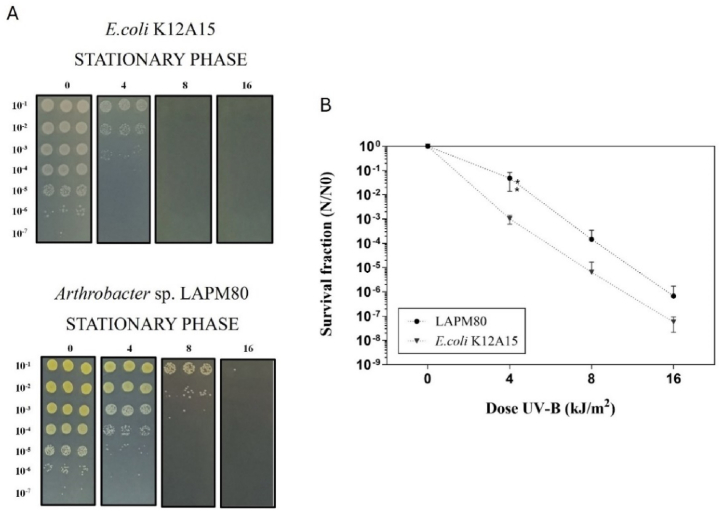
UV-B irradiation survival of *Arthrobacter* sp. strain LAPM80 and *E. coli* K12A15. (A) CFUs after exposing the strains in the stationary growth phase to 0, 4, 8, and 16 kJ/m^2^ doses of UV-B irradiation (312 nm). Bacteria were serially diluted (1:10^1^–1:10^7^) and dropped onto LB agar plates. (B) Survival fraction of the LAPM80 strain (●) and *E. coli* K12A15 (▼) in the stationary growth phase after exposure to the UV-B doses of irradiation (312 nm). Error bars represent the standard deviation of the three biological replicates. Asterisks indicate a significant difference (p < 0.05) between the survival of both isolates, assessed using a one-tailed Mann–Whitney significance test.

The survival fraction of the *Arthrobacter* sp. strain LAPM80, exposed to UV-B and UV-C irradiation, was compared to that of a reference strain, *E. coli* wild-type K12A15, which is proficient in DNA repair/tolerance systems [81]. In the case of UV-B irradiation, Fig. 5 shows that the *Arthrobacter* sp. strain LAPM80 tended to exhibit higher values than the reference strain, both in the stationary growth phase. However, for UV-C irradiation, both strains showed similar survival patterns (Fig. S3). Comparing these results with a recent study involving another Antarctic bacterium, the survival fractions of *Microbacterium* sp. LEMMJ01 (ornithogenic soil on King George Island) were significantly higher after exposure to UV-B and UV-C irradiation doses compared to the reference strain *E. coli* K12A15 [52]. Furthermore, after exposition to ∼8 kJ/m^2^ of UV-B irradiation, the *Arthrobacter* sp. strain LAPM80 exhibited moderate resistance with 4 logs of kill, while *Microbacterium* sp. LEMMJ01 had approximately 1 log of death [52]. Similarly, under exposure to doses between 100 and 120 J/m^2^ of UV-C irradiation, the *Arthrobacter* sp. strain LAPM80 exhibited moderate resistance with approximately 5 logs of kill, and *Microbacterium* sp. LEMMJ01 exhibited approximately 1 log of death [52].

Due to the intense solar radiation in Antarctica, regions such as King George Island may host bacteria resistant to UV radiation. Several studies have found correlations between solar radiation levels and the UV resistance of bacteria in certain environments. However, such correlations have not been consistent across other habitats. Nevertheless, many microorganisms isolated from diverse natural environments exhibit high resistance to UV radiation [13,52,76,[82], [83], [84]]. In addition, in some reports, UV resistance has been linked to taxonomic position [76,85], but interspecies variability in resistance may exist [84].

From the trains isolated from soil on King George Island (Antarctic), *Microbacterium* sp. LEMMJ01 was selected for its high resistance to UV-B irradiation compared to *Arthrobacter psychrochitiniphilus*, *Planococcus* sp., *Bacillus amyloliquefaciens*, and *E. coli* K12A15 [52]. Also, despite being isolated from the same geographic region with similar UV incidence, *Microbacterium* (ornithogenic soil) and the pigmented *Arthrobacter* sp. strain LAPM80 (moss soil) exhibited variations in UV irradiation resistance. These differences may arise from variations in the responses of pigmented microorganisms to UV exposure, as well as differences in pigment structure, location within the membrane, and concentration [77]. Additionally, variability in UV resistance among strains may depend on cellular machinery factors, such as DNA repair mechanisms and response to oxidative stress [53]. Antarctic ornithogenic soil is often compared to open desert soil, particularly in terms of sky-exposed bacteria that are shaped by high solar irradiance. As a result, bacteria from these environments frequently exhibit greater tolerance to solar irradiation than those from temperate terrestrial environments [12,51,52,86,87].

The isolation and selection of radio-resistant microorganisms have led to the development of bioproducts with potential photoprotection applications in the cosmetic industry. One natural alternative for photoprotection includes microbial carotenoids, which have been extensively studied for their antioxidant and sunscreen activity, and their potential application in photoprotective formulations [52,[88], [89], [90]]. Furthermore, isolating carotenoids with excellent properties holds significance, given their potential for patentable applications [[91], [92], [93]]. Some bacterial carotenoids tested for potential use in photoprotective formulations include sarcinaxanthin (C50) from *Micrococcus luteus* [91]; a carotenoid extract containing neurosporene, alpha-carotene, equinonone, canthaxanthin, and astaxanthin from *Microbacterium* sp. LEMMJ01 [52,88]; and a probable carotene extracted from *Brevibacterium* sp [80]. Additionally, isorenieratene and dihidroxiisorenieratene (C40) from *Brevibacterium linens* have shown the potential to decrease UV damage in human cell fibroblasts and could serve as natural compounds for skin cancer prevention [94].

The carotenoid pigments found in the extract of the *Arthrobacter* sp. strain LAPM80 may contribute to survival under UV-B irradiation, apart from DNA repair mechanisms and other responses to oxidative damage that may counteract UV-B-induced damage. However, further tests are required to confirm the photoprotective properties of the extract, such as mixing it with a pigment-free culture to assess the extent of protection provided by the carotenoids. For example, a carotenoid extract from *Microbacterium* sp., LEMMJ01 (ornithogenic Antarctic soil containing alpha-carotene, echinenone, canthaxanthin, and astaxanthin) protected *E.coli* K12A15 from UV-B irradiation (312 nm) damage [52]. Similarly, extracts from pigments produced by *Flavobacterium* sp. (zeaxanthin) and *Brevibacterium* sp. (mangrove soil, Brazil) protected *E. coli* under UVA-B irradiation exposure [80]. Rarely, certain carotenoids appear to protect against UV-C irradiation such as red and yellow carotenoids from *Micrococcus roseus* and *Micrococcus luteus* (Savandurga hills area, India), respectively, provided photoprotection to S. *faecalis* against UV-C radiation [95]. Additionally, a carotenoid extract (predominantly beta-carotene) from *Methylobacter* sp. N39 (rice plant leaves) protected *E. coli* against UV irradiation (254 nm) exposure [96].

### 3.5 Chemical characterization of carotenoids

The total carotenoids presented in *Arthrobacter* sp*.* LAPM80 extract were characterized by HPLC-DAD and HPLC-DADMS/MS. HPLC-DAD analysis revealed six peaks with UV–VIS spectra characteristic of carotenoids (Table S4; Fig. S4). Typically, carotenoids exhibit a three-peak absorption spectrum, with maxima (*λ*_max_) falling within the 400–500 nm range. The *λ*_max_ and spectral shape vary depending on the number of conjugated double bonds and other structural features [97]. The major carotenoids found in the strain (Rt = 26.12 and 26.78 min) displayed nearly identical UV–VIS spectra (*λ*_max_ 416, 440, and 470), suggesting similar structures (Fig. S4).

**Fig. 6:**
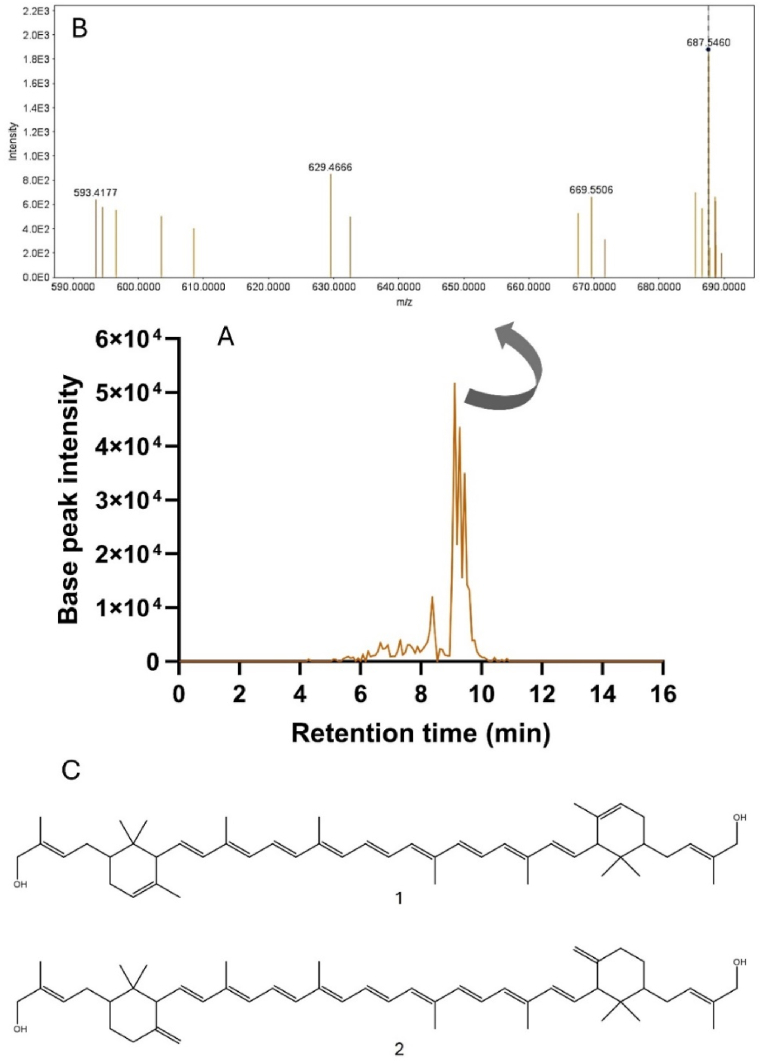
(A) Extracted ion chromatogram of *m*/*z* 705.5649 from MS full-scan data. (B) MS/MS spectra of ion 705.5649. (C) Structures of the carotenoids decaprenoxanthin (1) and sarcinaxanthin (2) proposedly present in the extract of the *Arthrobacter* sp. strain LAPM80.

For partial identification of carotenoids present in the LAPM80 strain, the extract underwent further analysis using HPLC-DAD-MS/MS. The region between 8 and 11 min showed peaks with absorption at 450 nm. Ions at this region were further examined. Notably, ions with *m*/*z* 705.5649 [M+H]^+^ and a likely molecular formula of C_50_H_73_O_2_ (calculated mass = 705.5611; error = 5.45 ppm) stood out (Fig. 6A). Fragmentation of this ion yielded multiple peaks, including 687 [M + H-18] and 669 [M + H-18-18], which indicated the loss of water. Additionally, there was a peak at 595 [M + H-18-92], corresponding to the elimination of water and a unit of toluene. These findings suggest the presence of carotenoids with 50 carbons in the sample under study.

According to previous literature and a carotenoid database [98], the C50 carotenoids decaprenoxanthin, sarcinaxanthin, and okadaxanthin share the molecular formula C_50_H_72_O_2_ and exhibit UV/Vis spectra similar to the major carotenoids detected in the extract of the LAPM80 strain [60,99,100]. Okadaxanthin [99] was previously isolated from species of the genus *Pseudomonas*, while decaprenoxanthin and sarcinaxanthin were identified in bacteria from the genus *Arthrobacter* (Fig. 6B) [60,100]. Thus, decaprenoxanthin and/or sarcinaxanthin are likely structures among the major carotenoids produced by the *Arthrobacter* sp. strain LAPM80. However, during the HPLC-DAD-MS/MS analysis, multiple peaks with a mass of 705 were observed, indicating the presence of different isomers. Therefore, carotenoids with structures not previously described may be present in the pigment extract as minor components. The confirmation of the structure of LAPM80 major carotenoids and the identification of minor compounds require comparison with standards, or their purification and isolation followed by analysis using other techniques such as nuclear magnetic resonance.

Although most natural carotenoids have 40 carbon atoms (C40), few possess 50 carbon atoms (C50). C50 carotenoids, primarily produced by bacteria and archaea, have similarities like having multiple conjugated double bonds, are synthesized from lycopene and contain at least one hydroxyl group, exhibiting strong antioxidant activity [69,100,101]. C50 carotenoids such as bacterioruberin, sarcinaxanthin, decaprenoxanthin, and arthroxanthin are reported in the literature due to their antioxidant properties [90,102], however differences between them are that an ε-ionone ring is found in decaprenoxanthin, an γ-ionone ring in sarcinaxanthin, four hydroxyl groups in bacterioruberin, and keto groups in arthroxanthin [58,103]. C50 carotenoid production of bacterial *Arthrobacter* species isolated from diverse environmental sources has been reported. For instance, *Arthrobacter glacialis* has been described to biosynthesize decaprenoxanthin, bisanhydrobacterioruberin, and Ag470 [104]. *Arthrobacter* sp. P40, and *Arthrobacter psychrochitiniphilus* produced decaprenoxanthin and its derivatives [12,60]. Decaprenoxanthin, sarcinaxanthin, and their derivatives were also detected in the pigment extract of *Arthrobacter arilaitensis* [100]. Additionally, *Arthrobacter agilis, Arthrobacter* sp. NamB2, and *Arthrobacter bussei* produced bacterioruberin and its derivatives [12,74,105], while arthroxanthin was synthesized by *Arthrobacter* sp. QL17 [58].

C50 carotenoids present in the extract of the *Arthrobacter* sp. strain LAPM80 may be responsible for the observed UV-B irradiation resistance. Additionally, the LAMP80 extract may exhibit photoprotective properties against oxidative damage induced by UV-B irradiation and thus has biotechnological applications. This study is the first to report that a possibly novel strain of *Arthrobacter*, isolated from the rhizosphere soil of King George Island (Antarctic Peninsula), produces C50 carotenoids (decaprenoxanthin and/or sarcinaxanthin) with potential photoprotective activity. The importance of C50 carotenoids in sunscreens is underscored by their potential commercial applications. For instance, sarcixanthin and its derivatives are included in patent applications as components of photoprotective formulations. This pigment was isolated from a novel strain, *Micrococcus luteus* Otnes 7 (a surface microlayer at the mid-region of the Norwegian Coast), which produced higher carotenoid quantities than other known strains at the time [91]. Similarly, decaprenoxanthin produced by *Arthrobacter psychrochitiniphilus* 366 (defrost biofilm, Antarctic), bacterioruberin synthesized by *Arthrobacter agilis* 50cyt (shells found in bird nests, Antarctica), and bacterioruberin from *Halorubrum* sp. HRM-150 (brine water) holds potential as natural components of sunscreens due to their antioxidant activity [12,102]. Additionally, a novel C50 carotenoid arthroxanthin, isolated from *Arthrobacter* sp. (Mount Qomolangma, Tibet), has potential application in the pharmaceutical industry due to its antioxidant activity [58]. Carotenoid activity mentioned before is interesting because protective mechanisms are crucial for mitigating the damage of UV-B solar radiation [13], being that UV-B rays have raised concerns, as they are mainly responsible for sunburns and skin cancer [106,107]. Thus, bacterial carotenoids hold promise for use in sunscreens, addressing the current demand for natural and environmentally friendly products in the cosmetics sector [108,109]. Moreover, C50 carotenoids are suitable for cosmetics due to their solubility and stability in oily emulsions [103].

### 3.6 Scanning electron microscopy analysis

**Fig. 7:**
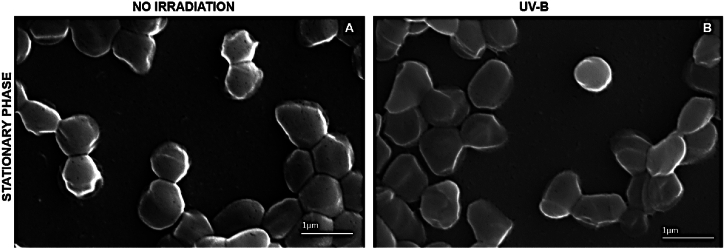
Scanning Eletron Microcopy (SEM) images of *Arthrobacter* sp. LAPM80 cells in the stationary growth phase, after exposure to 6 kJ/m^2^ of UV-B irradiation (B) compared to nonirradiated samples (A), serving as the control. Image magnification: 45000 ×. Representative fields were chosen to illustrate the main cellular modifications observed.

SEM micrographs depicted the cell morphology of the *Arthrobacter* sp. strain LAPM80 after exposure to UV-B irradiation (312 nm) in comparison to nonirradiated samples from the logarithmic and stationary growth phases (Figs. S5A, S5B, 7A and B). The images revealed that nonirradiated cells exhibited rod shapes with smooth surfaces at the logarithmic growth phase (Fig. S5A) and pleomorphic forms at the stationary growth phase (Fig. 7A). Furthermore, the micrographs showed surface irregularities and aggregation in some cells of the logarithmic growth phase (characterized by low pigment content) after exposure to 4 kJ/m^2^ of UV-B irradiation (Fig. S5B). However, no significant changes were observed in the bacterial cell structure of the stationary growth phase (characterized by high pigment content) when exposed to 6 kJ/m^2^ of UV-B irradiation (Fig. 7B), with only slight deformations noted at the cell surface. This cell surface protection could be attributed to the high pigment concentration in the bacteria during the stationary growth phase.

There are no reports of SEM micrographs depicting *Artrhobacter* from the Antarctic following UV irradiation exposure. However, SEM micrographs of another Antarctic *Actinomycetota*, *Microbacterium* LEMMJ01 (ornithogenic soil) showed no morphological alterations in cells in the stationary growth phase exposed to UV-B irradiation (312 nm) [52]. In contrast, the UV-B-sensitive type strain *E. coli* K12A15 exhibited irregular membranes with surface leakage of intracellular material in SEM micrographs post-exposure to UV-B irradiation (312 nm) [52]. In a report of another *Actinomycetota* UV-resistant, *Nesterenkonia* sp. strain Act20 (soil around Lake Socompa, Argentina) and its control *Nesterenkonia halotolerans* DSM 15.474 were cultured until reached the logarithmic phase and then exposed to UV-B irradiation (312 nm). In the SEM micrographs, as the dose increased, the morphology of Act20 widened and lengthened due to the disruption of cell division, also cell surfaces exhibited signs of shrinkage. In *N. halotolerans*, fibrillar structures were observed solely on the surfaces, thick pili or disintegrated structures were noted, cell aggregates were formed due to the production of extracellular polymeric substances, and surface irregularities indicating cell-wall degradation and rupture leading to cell lysis were observed [110]. In summary, UV-B irradiation could induce membrane alterations or cell aggregation, with more pronounced effects observed in the cells of the *Arthrobacter* sp. strain LAPM80 harvested during the logarithmic growth phase, characterized by low pigment production.

## 4 Conclusion

The condition of high UV radiation in the Antarctic could influence the establishment of UV-resistant microorganisms, being a rich source of pigmented bacteria. It was aimed to study the carotenoid production capability of *Arthrobacter* and the UV irradiation resistance as influenced by the presence and concentration of carotenoid in their cells. We assessed a possible novel strain, *Arthrobacter* sp. LAPM80, from moss soil. This strain exhibited superior production of total carotenoids, including rare C50 carotenoids (primarily decaprenoxanthin, as suggested by genomic annotation). Adittionally, preliminary tests indicated that the high carotenoid concentration in LAPM80 during its stationary phase likely enhances its UV-B irradiation resistance.

This study advances our understanding of the potential photoprotective effects of rare C50 carotenoids in a possibly new *Arthrobacter* species from the moss rhizosphere of King George Island, Antarctic Peninsula. It also lays the groundwork for further investigations into the capacity of bacterial strains isolated from rhizospheric soils of King George Island to produce photoprotective C50 carotenoids, which could have potential applications in the cosmetics industry.

## CRediT authorship contribution statement

**Beatriz Vivian Paredes Contreras:** Writing – review & editing, Writing – original draft, Validation, Investigation, Formal analysis, Data curation, Conceptualization. **Alane Beatriz Vermelho:** Writing – review & editing, Supervision, Resources, Project administration, Funding acquisition, Formal analysis, Conceptualization. **Livia Casanova:** Writing – review & editing, Methodology, Formal analysis. **Claudia de Alencar Santos Lage:** Writing – review & editing, Validation, Resources, Investigation, Formal analysis. **Caren Leite Spindola Vilela:** Writing – review & editing, Writing – original draft, Visualization, Investigation, Formal analysis. **Veronica da Silva Cardoso:** Writing – review & editing, Validation, Methodology, Formal analysis. **Luis William Pacheco Arge:** Methodology, Formal analysis. **Janine Simas Cardoso-Rurr:** Methodology, Formal analysis. **Sulamita Santos Correa:** Methodology, Formal analysis. **Felipe Raposo Passos De Mansoldo:** Methodology, Formal analysis, Data curation. **Maria Cristina Pinheiro Pereira Reis-Mansur:** Writing – review & editing, Investigation, Formal analysis, Data curation. **Eikon Alves da Silva:** Investigation, Formal analysis. **Júnia Schultz:** Writing – review & editing, Writing – original draft, Supervision, Formal analysis, Data curation, Conceptualization. **Alexandre Soares Rosado:** Writing – review & editing, Supervision, Resources, Project administration, Funding acquisition, Conceptualization.

## Data availability

The 16S rRNA gene and the draft genome sequence of the strain LAPM80 characterized in this study were deposited in NCBI under BioProject PRJNA1103223. The 16S and WGS accession numbers are available in Table S2; the genome version described in this paper is the first.

## Funding

This work was supported by the Coordination for the Improvement of Higher Education Personnel (10.13039/501100002322CAPES), Brazil. ASR was financed by KAUST Baseline Grant (BAS/1/1096-01-01), Saudi Arabia.

## Declaration of competing interest

The authors declare that they have no known competing financial interests or personal relationships that could have appeared to influence the work reported in this paper.
