# Supplementary material for: Enhanced UV-B photoprotection activity of carotenoids from the novel Arthrobacter sp. strain LAPM80 isolated from King George Island, Antarctica
Source: Heliyon. 2024 Dec 21;11(1):e41400. doi: 10.1016/j.heliyon.2024.e41400 (PMC11731199; doi:10.1016/j.heliyon.2024.e41400)
Supplement: Multimedia component 1 [file mmc1.docx]

**SUPPLEMENTARY MATERIALS**

**Table S1**. Codes and isolation sites of *Actinomycetota* strains collected from the Antarctic Bacterial Collection of LEMM used in this study.

| **Strain code** | **Isolation place** |
| --- | --- |
| LAPP5, LAPP35, LAPP59, LAPP65, LAPP68, and LAPP70 | Ornithogenic soil |
| LAPM78, LAPM80, and LAPM173 | Rhizosphere of moss |
| LAPYS85 and LAPYS90 | Yellow soil |
| LAPC111, LAPC166, LAPC215, LAPC314, LAPC319, and LAPC320 | Rhizosphere of *Colobanthus quitensis* |
| LAPD122, LAPD123, and LAPD300 | Rhizosphere of *Deschampsia antarctica* |

**Table S2**. Summary of the draft genome assembly and annotation metrics.

| **Parameters** | **STRAIN LAPM80** |
| --- | --- |
| Total length | 4034042 |
| Number of contigs | 68 |
| GC% content | 62.80 |
| N50 (bp) | 202837 |
| Largest contig (bp) | 511798 |
| Coding sequences | 3929 |
| tRNA | 54 |
| rRNA | 4 |
| ANI | ~81.92% |
| dDDH | ~23.1% |
| Complete genes (BUSCO) | 97.6% |
| ChecKM_completeness | 100 |
| ChecKM_contamination | 0.6 |
| Top identification | *Arthrobacter sp.* |
| Isolation source | Moss rhizosphere |
| 16S accession number | PP708905 |
| WGS accession number | SUB14395447 |

**Table S3.** Enzymes related to the carotenoid synthesis pathway according to Diamond BLASTp.

| **NCBI id** | **Description** | **Identities** | **Positives** |
| --- | --- | --- | --- |
| MDD0858641.1 | Phytoene/squalene synthase family protein | 204/291 (70%) | 227/291 (78%) |
| WP_231687086.1 | Phytoene desaturase family protein | 426/587 (73%) | 477/587 (81%) |
| ALV47569.1 | Phytoene dehydrogenase | 394/536 (74%) | 438/536 (82%) |
| MDD0858640.1 | Lycopene cyclase domain-containing protein | 80/107 (75%) | 89/107 (83%) |
| MEC5170668.1 | Zeaxanthin glucosyltransferase | 299/427 (70%) | 341/427 (80%) |
| RAN71827.1 | Geranylgeranyl pyrophosphate synthase | 303/341 (89%) | 325/341 (95%) |
| ALE94134.1 | C50 carotenoid epsilon cyclase | 72/99 (73%) | 81/99 (82%) |

**Table S4.** HPLC-DAD analysis of the carotenoid extract of *Arthrobacter* sp. LAPM80 and proposed structures; peaks were detected at 450 nm.

| **Peak** | **Retention time (min)** | **λ_max_ (nm)** | **Relative area (%)** | **Proposed structure** |
| --- | --- | --- | --- | --- |
| 1 | 22.38 | 416 | 5.8 | Not identified |
| 2 | 22.71 | 422 | 9.4 | Not identified |
| 3 | 26.12 | 416, 440, and 470 | 48.2 | Carotenoid |
| 4 | 26.78 | 416, 440, and 470 | 22.5 | Carotenoid |
| 5 | 27.50 | 418, 442, and 470 | 0.8 | Carotenoid |
| 6 | 27.70 | 414, 436, and 466 | 2.8 | Carotenoid |
| 7 | 28.14 | 412, 426, and 464 | 4.2 | Carotenoid |
| 8 | 28.65 | 412, 434, and 462 | 1.3 | Carotenoid |


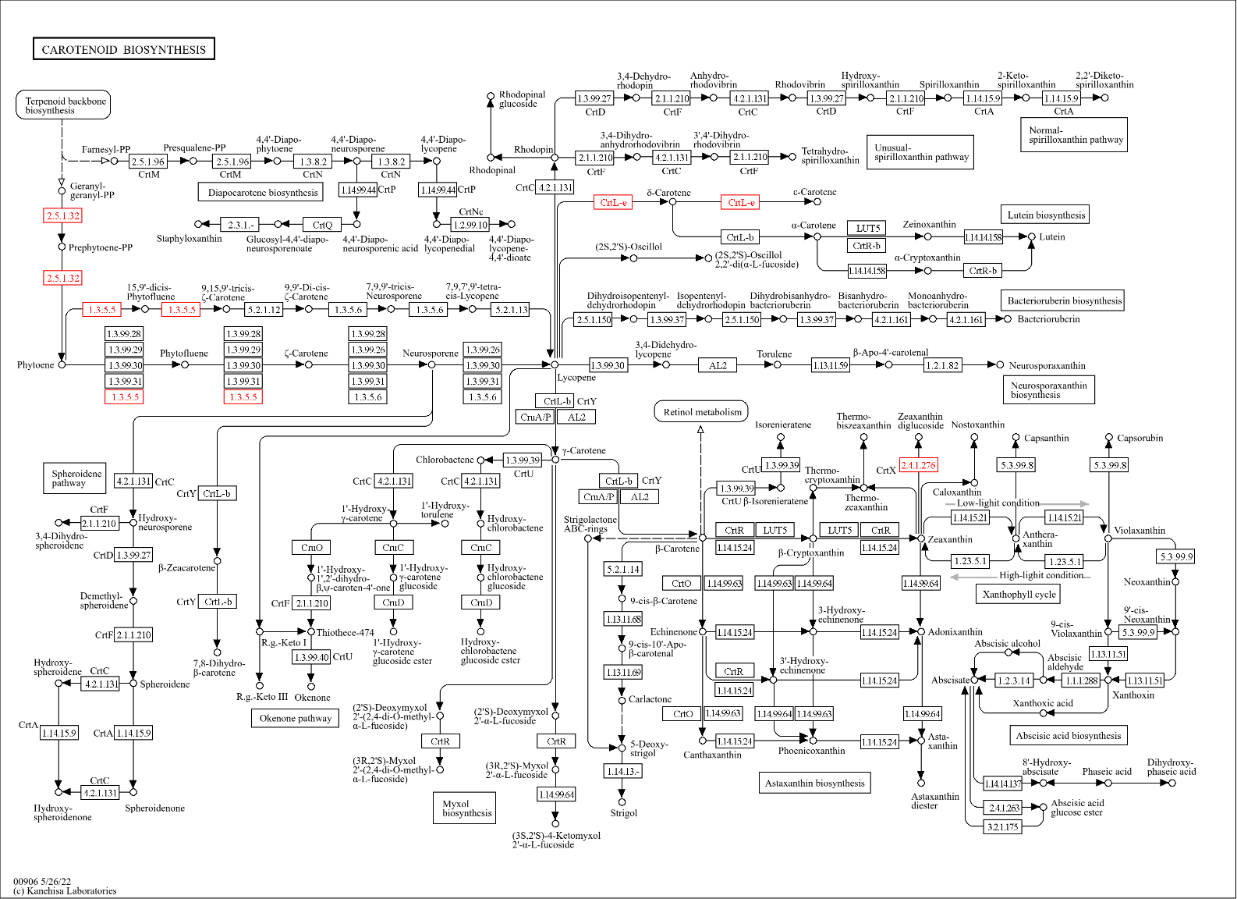


Figure S1. Diagram of the synthesis of carotenoids from KEGG. The enzymes found in this work are highlighted in red, which are phytoene synthase (K02291), phytoene desaturase (K02293), lycopene cyclase (K06444), zeaxanthin glucosyltransferase (K14596), geranylgeranyl pyrophosphate synthase (K13787).


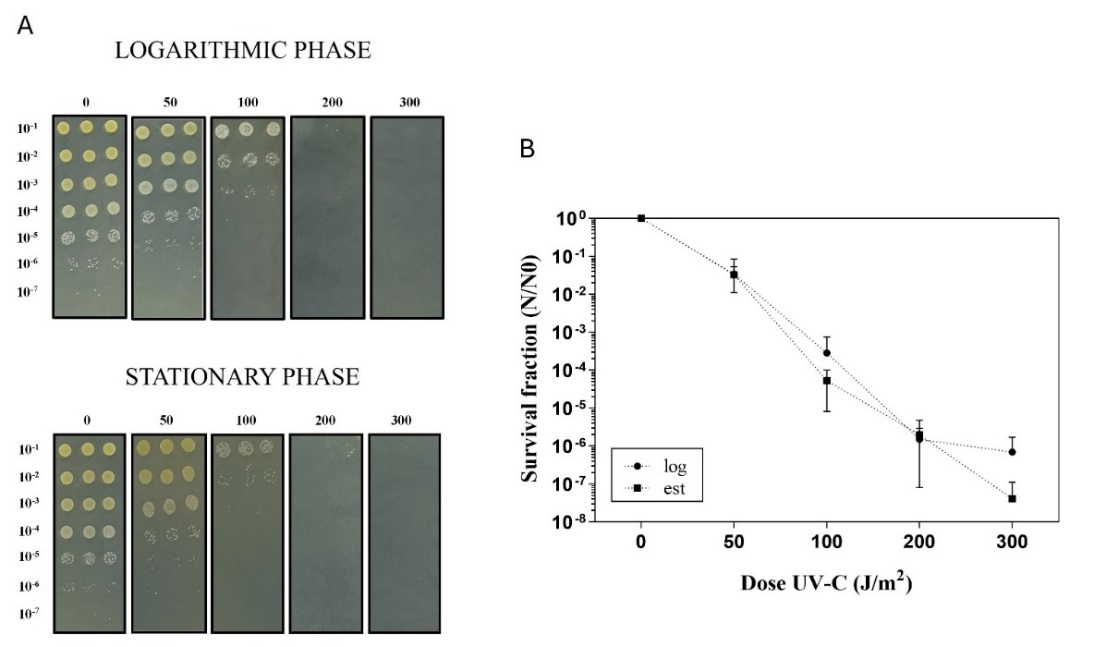


**Figure S2.** UV-C irradiation survival of *Arthrobacter* sp. strainLAPM80. (A) CFUs after exposing the strain in the growth phases to 0, 50, 100, 200, and 300 J/m^2^ of UV-C irradiation doses (254 nm). Bacteria were serially diluted (1:10^1^–1:10^7^) and dropped onto LB agar plates. (B) Survival fraction of LAPM80 strain in the logarithmic phase (●) and stationary phase (■) after exposure to the UV-C doses of irradiation. Error bars represent the standard deviation of three biological replicates. Asterisks indicate a significant difference (p < 0.05) between the survival of both growth phases, assessed using a one-tailed Mann–Whitney significance test.


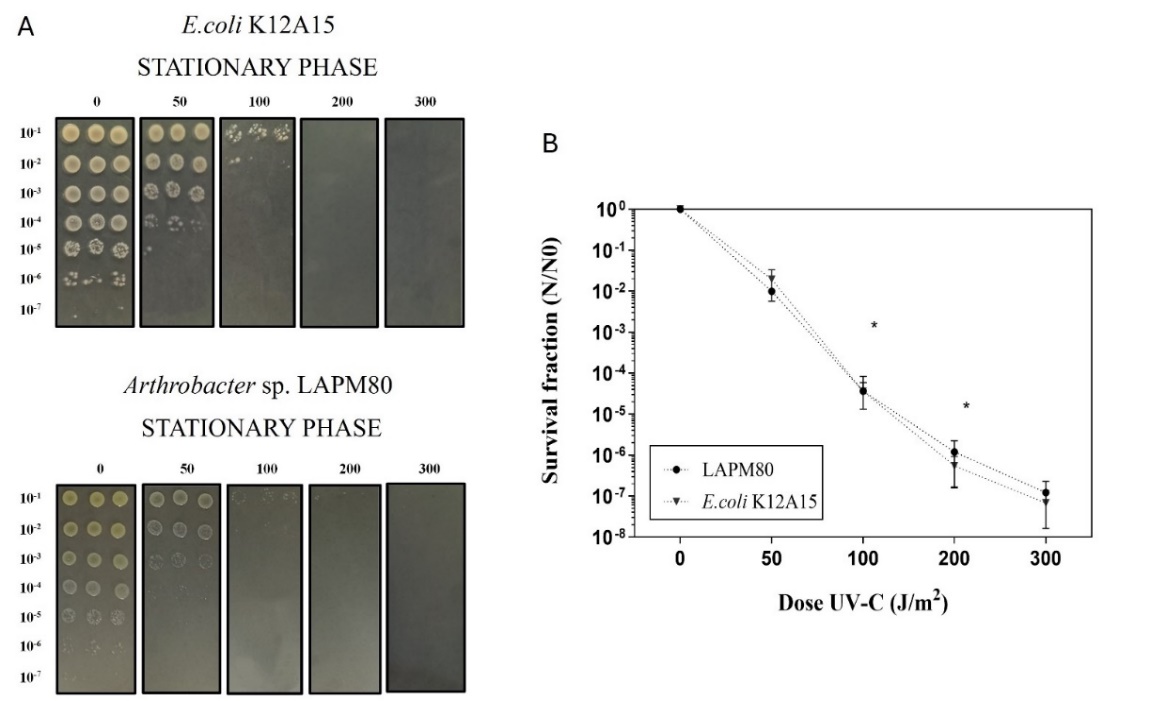


**Figure S3**. UV-C irradiation survival of *Arthrobacter* sp. strain LAPM80 and *E. coli* K12A15. (A) CFUs after exposing the strains in the stationary growth phase to 0, 50, 100, 200 e 300 J/m^2^ of UV-C irradiation doses (254 nm). Bacteria were serially diluted (1:10^1^–1:10^7^) and dropped onto LB agar plates. (B) Survival fraction of LAPM80 strain (●) and *E. coli* K12A15 (▼) in the stationary growth phase after exposure to the UV-C doses of irradiation (254 nm). Error bars represent the standard deviation of the three biological replicates. Asterisks indicate a significant difference (p < 0.05) between the survival of both isolates, assessed using a one-tailed Mann–Whitney significance test.

Figure S4. HPLC-DAD chromatogram (450 nm) of the carotenoid extract of *Arthrobacter* sp. LAPM80. UV spectra of the major peaks are depicted.


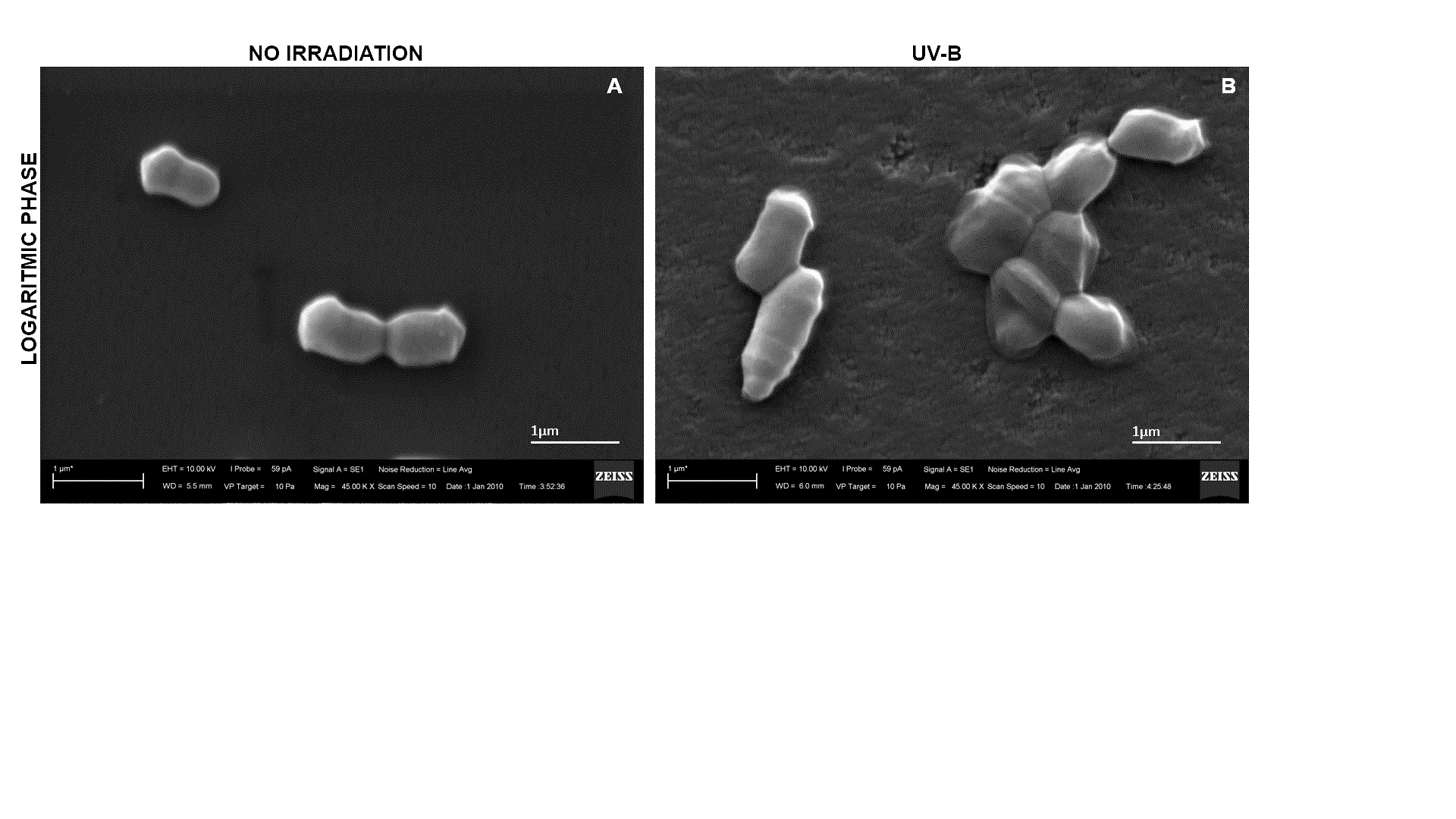


**Figure S5**. SEM images of *Arthrobacter* sp. LAPM80 cells, in the logarithmic growth phase, after exposure to 4 kJ/m^2^ of UV-B irradiation during the logarithmic growth phase (B) compared to nonirradiated samples (A), serving as the control. Image magnification: 45000× for A and B. The most representative images illustrating the main modifications were selected after observing all fields.
